# Early Screening for Developmental Language Disorder: Predictive Validity of German Parent Reports

**DOI:** 10.1111/1460-6984.70303

**Published:** 2026-08-04

**Authors:** Eveline Pinstock, Satyam Antonio Schramm

**Affiliations:** ^1^ Department of Inclusive Education University of Potsdam Germany

**Keywords:** developmental language disorder, DLD, Germany, parent report, predictive validity, screening

## Abstract

**Background:**

Developmental Language Disorder (DLD) is a prevalent neurodevelopmental condition associated with long‐term cognitive, academic, and psychosocial consequences. Early identification is critical for timely intervention, yet predictive frameworks within German‐speaking contexts remain limited. Parent‐report questionnaires such as the FRAKIS‐K and SBE‐2‐KT are widely used in the German paediatric U7 check‐up for two‐year‐olds, but their predictive validity for DLD has not been systematically evaluated.

**Aims:**

This study assessed the predictive validity of the FRAKIS‐K and SBE‐2‐KT administered at age two for identifying risk for DLD at age four. A secondary objective was to test whether adjusting the screening cut‐off values could enhance predictive accuracy.

**Methods and Procedures:**

A community sample of 500 families in Hanover, Germany, was contacted through the city register; 186 children (mean age = 2;0) participated at the first assessment. At age four (*N* = 145), expressive vocabulary and grammar were measured using the Potsdam‐Illinois Test of Psycholinguistic Abilities (P‐ITPA). Children scoring ≤ –1.5 SD on P‐ITPA subtests were classified as having language impairment consistent with risk for DLD. Predictive validity indices—sensitivity, specificity, positive and negative predictive values, relative improvement over chance (RIOC), and area under the curve (AUC)—were calculated for both parent‐report tools. ROC analysis was applied to explore optimal cut‐off adjustments.

**Outcomes and Results:**

Prevalence of language impairment at age four was 5.5%. The SBE‐2‐KT showed moderate predictive power (*AUC* = 0.85; *RIOC* = 0.51), with sensitivity of 57% and specificity of 89%. The FRAKIS‐K vocabulary alone performed similarly (*AUC* = 0.83; *RIOC* = 0.58), achieving sensitivity of 63% and specificity of 91%, while including grammar criteria reduced predictive validity. ROC analysis indicated improved sensitivity (up to 71%) with minimal specificity loss when adjusting cut‐offs.

**Conclusion and Implications:**

Both FRAKIS‐K vocabulary and SBE‐2‐KT demonstrate adequate predictive validity for identifying risk of DLD at age two within the German U7 screening context. They function effectively as first‐tier tools to rule out children not at risk, though positive screens should be followed by comprehensive assessments. The study's longitudinal design and evaluation of clinically relevant screening instruments using multiple predictive validity indices strengthen the findings. While refining vocabulary‐based cut‐offs may improve early detection, the small number of children with language impairment in the sample limits the generalizability of optimization suggestions, highlighting the need for further research in larger clinical populations.

**WHAT THIS PAPER ADDS:**

*What is already known on this subject*

- Parent‐report questionnaires are commonly used for early language screening, but their predictive validity for DLD is often limited. In German‐speaking contexts, evidence on the predictive accuracy of brief tools such as FRAKIS‐K and SBE‐2‐KT is scarce.

*What this study adds to the existing knowledge*

- This longitudinal study shows that both FRAKIS‐K (vocabulary component) and SBE‐2‐KT at age two moderately predict outcomes of language impairment at age four. Adjusting the screening cut‐offs increased sensitivity while maintaining acceptable specificity, indicating that predictive validity may be improved through threshold refinement. However, as the sample included few children with risk for DLD, recommendations for optimizing these tools should be interpreted with caution.

*What are the potential or actual clinical implications for this work?*

- FRAKIS‐K vocabulary and SBE‐2‐KT can be used as first‐tier screening instruments during the U7 paediatric check‐up in Germany. Using adjusted cut‐offs may enhance early identification of children at risk for DLD, supporting earlier referral and intervention, though further validation in larger samples is required.

## 1 Introduction

Developmental language disorder (DLD) is a significant language impairment that affects daily functioning, has a negative prognosis, and lacks a clear biomedical aetiology (Bishop et al. 2017). Children with DLD are at risk for co‐occurring cognitive, emotional, and social difficulties, as well as poor educational outcomes (Bleses et al. 2016) and increased psychological distress (Lee et al. 2020). Longitudinal studies indicate that DLD does not resolve spontaneously, and persistence into school age often leads to further developmental impairments (Snowling et al. 2001).

DLD is a common neurodevelopmental disorder with international prevalence estimates ranging widely depending on diagnostic criteria. While rates vary from 2.5% in Finland (Hannus et al. 2009) to 19.7% in Egypt (Gad‐Allah et al. 2012), most studies converge on approximately 7%–8%, including in the U.S., U.K., China, and Mexico (Tomblin et al. 1997; Norbury et al. 2016; Wu et al. 2023; Auza et al. 2024). Its frequency and impact have increased attention to diagnostic standards, early intervention, and preventive strategies.

Early identification of DLD in German‐speaking populations remains under development. This study examines the predictive validity of the German parent questionnaires FRAKIS‐K (Szagun et al. 2009, 2023) and SBE‐2‐KT (Suchodoletz and Sachse, 2009) for two‐year‐old children, using standardized language assessment at age four as the outcome, while also exploring the potential for optimizing cut‐off values.

The following section reviews briefly on early screening practices in Germany, including parent‐report tools FRAKIS‐K and SBE‐2‐KT, discusses the gold standard for DLD diagnosis, and synthesizes research on the predictive validity of parent reports for early language abilities.

### 1.1 Early Screening Practices in Germany and the Use of FRAKIS‐K and SBE‐2‐KT

In Germany, all toddlers undergo annual paediatric health check‐ups (BVKJ 2024). Around the second birthday, the U7 examination includes an assessment of language development, though paediatricians choose the evaluation method. Two widely used parent‐report questionnaires are FRAKIS‐K and SBE‐2‐KT (AWMF 2022). FRAKIS‐K comprises a 102‐word checklist and three grammar items assessing word combinations, plural formation, and determiners, while SBE‐2‐KT features a 57‐word checklist and one item on word combinations. Their relatively short word lists make both tools practical and ecologically valid for the U7 setting. Parent‐questionnaire screening is considered a first step in the diagnostic process, with further monitoring or evaluation initiated if a child shows limited language proficiency (AWMF 2022).

### 1.2 No Gold Standard for Diagnosing DLD

A discussion regarding the gold standard for the diagnosis of DLD is ongoing. To date, there is no established procedure for diagnosing DLD. In CATALISE‐2, a Delphi consensus study on terminology concerning DLD in English‐speaking countries, a consensus was reached that prognosis is a key diagnostic criterion (Bishop et al. 2017). However, the criteria for categorizing a child's language problems as being ‘unlikely to resolve without specialist help’ are unclear. The consensus for German‐speaking countries was based on the Delphi study by Lüke et al. (2023). Different professions agree that a ‘significant deviation from typical language development’ is relevant for the diagnosis of DLD. In particular, a child's score on a standardized test should be at least 1.5 standard deviations (SD) below the mean for children of the same age. Whether a single subtest score alone justifies the diagnosis of a disorder remains controversial. Furthermore, the earliest age for diagnosing DLD in German‐speaking countries is three years (Lüke et al. 2023). In comparison, DSM‐5 suggests diagnosing language disorders beginning at the age of four. At this age, individual differences in language skills are more stable, can be measured more accurately with assessment instruments, and are good predictors of later language skills (APA 2013).

Importantly, screening should be distinguished from diagnosis. Evidence summarized by Sansavini et al. (2021) indicates that language abilities starting at the age of two years are already informative for later outcomes, supporting screening at this age despite later diagnostic thresholds.

Nonetheless, early identification of DLD remains challenging because language development in early childhood is highly heterogeneous. Although many late talkers catch up to their peers (Pinstock and Schramm 2026), some go on to develop persistent language disorder (Rescorla 2011), while others with DLD do not exhibit clear delays during the toddler years (Snowling et al. 2016). Moreover, the relatively low prevalence of DLD creates inherent challenges for early screening, as efforts to increase case detection may also increase the number of false‐positive identifications (Klee et al. 2000). These developmental patterns have prompted considerable research into whether early parent‐report measures can reliably distinguish children at risk for later language difficulties.

### 1.3 Predictive Validity of Parent Reports

A substantial body of research has investigated the extent to which parent questionnaires can predict later language outcomes. Overall, findings suggest that these measures often demonstrate high specificity, whereas sensitivity tends to vary depending on methodological and developmental factors. Parent questionnaires tend to perform well in ruling out children without later language difficulties. For example, the MacArthur‐Bates Communicative Development Inventories (CDI), the Language Development Survey (LDS), and the Swedish Communication Screening at 18 months (SCS18) have all demonstrated specificities ranging from 87% to 95% in predictive screenings (Feldman et al. 2005; Heilmann et al. 2005; Klee et al. 1998; Westerlund et al. 2006). These values suggest that parent‐report tools are particularly effective in minimizing false positives.

In contrast, reported sensitivities vary more widely. Tools such as the CDI have shown high sensitivity in predictive screenings when administered at 24 months, with language outcomes assessed at 30 months (e.g., 81% in Heilmann et al. 2005), whereas less structured or earlier assessments yield substantially lower values. For example, Lieberman et al. (2022) reported a sensitivity of only 8% using open‐ended parental questions about babbling at 10 months to predict language screening outcomes at two‐and‐a‐half to three years. Sensitivity tends to decline in predictive screening with stricter diagnostic thresholds in language outcomes (e.g., ‐2 SD or 5th percentile) and increase with more lenient ones (Dale et al. 2003; Stott et al. 2002; Van Noort‐Van der Spek et al. 2021). These results reflect the familiar trade‐off in early screening between capturing as many true positives as possible and avoiding over‐identification.

Early language assessment is particularly focused on expressive vocabulary, which consistently emerges as a strong predictor of later language outcomes. Recent evidence further indicates that delays in expressive and receptive vocabulary, gesture production, syntactic comprehension, and early word combinations are among the most robust early indicators associated with later DLD (Sansavini et al. 2021). Vocabulary size in toddlerhood is reliably associated with subsequent expressive and receptive language skills and grammatical development (Bleses et al. 2008; Dale et al. 2003; Eadie et al. 2022). At age two, children with language delay typically produce only a few words and have not yet begun to use grammar productively (Grimm and Schulz 2014). Because formal testing at this age is time‐consuming and no more accurate than parent reports, parental questionnaires provide a practical and valid alternative (Fenson et al. 1994; Sachse and Suchodoletz 2008). These tools offer ecologically valid insights into children's language use, facilitating earlier identification and intervention (Auza et al. 2023; Neumann et al. 2024). Vocabulary acquisition patterns are relatively similar across languages such as English and German (Grimm and Schulz 2014), whereas grammar shows greater cross‐linguistic variability and requires language‐specific assessment.

Parent‐reported vocabulary size between 18 and 24 months is reliably associated with later expressive and receptive language skills. Westerlund et al. (2006) found that producing fewer than 8 words out of 90 in the SCS18 predicted language problems at age three with an area under the curve (AUC) of 0.77. Similarly, the CDI and LDS—both focused on expressive vocabulary—have repeatedly shown significant associations with later diagnostic outcomes (Klee et al. 1998; Feldman et al. 2005). More recently, Visser‐Bochane et al. (2021) demonstrated that a brief language screening protocol administered at age two achieved good predictive validity for language outcomes at age three (sensitivity = 0.82, specificity = 0.74), supporting the clinical utility of structured early screening in routine preventive healthcare settings.

Beyond vocabulary, instruments that assess multiple language domains—including pragmatics, grammar, and parental concern—often show higher predictive accuracy, especially in children over age two. Pesco and O'Neill (2012) demonstrated that the Language Use Inventory (LUI), which includes pragmatic items, achieved 81% sensitivity and 93% specificity (*AUC* = 0.91) in predicting later language outcomes at ages five to six years from assessments conducted at 24–47 months. This multidimensional structure enables broader coverage of language development and may be particularly useful when screening for more subtle or complex language impairments.

A growing number of studies address the cross‐linguistic and cultural validity of parent‐report tools. Recent work by Auza et al. (2023) in a Spanish‐speaking sample demonstrated that a culturally adapted parent questionnaire could effectively identify children with DLD, achieving predictive metrics comparable to those in English‐speaking samples. The editorial by Ezeizabarrena and Kovacevic (2024) highlights that when linguistically and culturally adapted, parent‐report tools are reliable across diverse language contexts. This is particularly important for screening in bilingual or minority‐language populations, where language development trajectories may differ from monolingual norms.

Several studies suggest that combining parent reports with professional observation or direct assessment improves predictive validity. In the Generation R study, Henrichs et al. (2011) found that parent‐reported vocabulary at 18 months (via the CDI Dutch version) predicted later language scores at 30 months on the LDS with high specificity (93%) but low sensitivity (30%), highlighting the need for complementary data sources. de Koning et al. (2004) similarly found that expert judgment yielded higher sensitivity (52%) than parent report alone (24%) in identifying language difficulties in Dutch toddlers screened at 18 and 24 months, with outcomes assessed at 36 months, although both methods demonstrated excellent specificity. In line with these findings, Holzinger et al. (2022) showed that a two‐stage screening approach at 24 months—where only children who screened positive on the parent‐reported questionnaire received an additional brief direct assessment of word comprehension—substantially improved predictive accuracy (sensitivity = 74%, specificity = 86%) for language outcomes at age three, compared to parent report alone, underscoring the benefits of integrating multiple data sources.

German parent reports such as the ELFRA‐2 (Grimm and Doil 2000) and SBE‐2‐KT, typically administered around 24 months, assess expressive vocabulary and emerging grammatical skills. In one study, Sachse and Suchodoletz (2008) examined the predictive validity of the ELFRA‐2 using a follow‐up at 37 months with the clinician‐administered standardized test SETK‐3‐5 (Grimm 2015), which includes subtests for both expressive and receptive language. They reported a sensitivity of 61% and specificity of 94%, suggesting strong predictive accuracy, particularly in identifying children without later language delays. The relatively high positive predictive value (PPV) also indicated that many of the children flagged at age two went on to show persistent difficulties. Ullrich and Suchodoletz (2011) further evaluated the ELFRA‐2 and SBE‐2‐KT using follow‐up data from the parent‐report SBE‐3‐KT (Suchodoletz et al. 2009) at 36 months. While the use of a parent‐report follow‐up introduces the possibility of shared method variance, both tools yielded high specificity (94%) and negative predictive value (NPV; 91%), indicating they were effective at ruling out children not at risk. Sensitivity was moderate at 46%, and PPV ranged from 56% to 59%. Although the high specificity and NPV in these findings make ELFRA‐2 and SBE‐2‐KT suitable for first‐level screening, their lower sensitivity and PPV indicate they should not be used in isolation for diagnostic decision‐making.

### 1.4 Current Study

Since DLD is a prevalent and persistent condition with significant developmental, educational, and psychosocial consequences, early identification is crucial. However, prediction in toddlerhood remains challenging due to heterogeneity in language development and the absence of a gold‐standard diagnostic framework. In Germany, routine paediatric screenings at age two provide an opportunity for early detection using parent report forms such as the FRAKIS‐K and SBE‐2‐KT. These brief questionnaires are used in clinical settings, but empirical evidence on their predictive validity remains limited. Existing research highlights a trade‐off between sensitivity and specificity in early screening, underscoring the need for carefully calibrated cut‐off scores. Therefore, this study evaluates the predictive validity of using FRAKIS‐K and SBE‐2‐KT at age two for identifying language impairment consistent with risk for DLD at age four. Subsequently, it explores whether modifying cut‐off values may enhance early screening performance. The findings aim to support the development of evidence‐based screening protocols within German paediatric care.

## 2 Methods

### 2.1 Participants

The participants were drawn from a community sample obtained from the city register in Hanover, Germany. The selected children belonged to an age group that included all those who would reach 18 months of age within the next two months. A total of 500 families were contacted by mail and were offered an incentive of five Euros for each completed questionnaire. Participants could choose to return the questionnaire by mail or complete it online. Of these, 219 (43.8%) agreed to participate in the study. As four of the participating families had twins, responses were received from the parents of 223 children. Twenty‐five children were excluded for the following reasons: 20 children did not speak German as their dominant language (for multilingual children, parents were asked whether German was the primary language spoken at home, because testing took place in German only), and four questionnaires could not be linked to the existing address list. After accounting for exclusion, the sample initially included 199 children aged 18 months in the broader study. Due to dropout over time, the sample size was 186 children when reaching the age of two, which we will refer to as the first assessment point in the current study. For clarity, we focus on the two assessment times that are relevant to this article. Due to additional dropout over time, 145 children participated in testing at four years of age. The dropout to 145 children disproportionately affected families with lower parental education. One more child appeared for testing at the last assessment point, but testing was not possible in this case because of behavioural difficulties. At the first assessment point, children were aged 2;0 on average, with an age range from 1;11 to 2;3. At the later assessment point, the children were aged 4;1, on average, with an age range of 3;10 to 4;2. More than half of the children (out of 145) were male (53.8%), and the majority (62.1%) were either first‐born or only children. Additionally, 18.6% of the participants were raised in bi‐/multilingual households where German was the dominant language. Furthermore, most of the children (80.7%) had at least one parent with a university degree.

### 2.2 Procedure

Early language abilities were assessed using two standardized parental report forms, FRAKIS‐K and SBE‐2‐KT.

The FRAKIS‐K, a short form of the German CDI, evaluates general expressive language ability. It includes a vocabulary checklist of 102 words and three grammar‐related questions (assessing article use, plural formation, and word combinations). Normative data is available for each month between 1;6 and 2;6. The authors of FRAKIS‐K (Szagun et al. 2009, 2023) categorize children's expressive vocabulary as ‘below the norm’ if it falls within the lowest percentile of the norm. Children with ‘below the norm’ vocabulary are referred to as late talkers. Within the context of U7 (two‐year paediatric check‐up in Germany), the authors urge to carefully consider the term late talker. They suggest that a two‐year‐old child is classified as a late talker if it meets all three criteria: (1) the expressive vocabulary falls within the lowest percentile, (2) plural forms are not yet used, and (3) word combinations are not yet used.

The SBE‐2‐KT was specifically designed to identify late talkers within the context of the U7. It includes a vocabulary checklist of 57 words and a single grammar‐related question that assesses word combinations. The response to this grammatical question is considered equivalent to knowing an additional word. Children are classified as late talkers if their expressive vocabulary falls within the lowest 14% of the normative sample. Separate normative data is available for children aged 1;9–1;10, and 1;11–2;0. Translations of SBE‐2‐KT are available in 34 languages; however, these versions have not yet undergone formal validation.

At age four, children underwent a standardized assessment of expressive language abilities using two subtests from the Potsdam‐Illinois Test of Psycholinguistic Abilities (P‐ITPA; Esser and Wyschkon 2010): one assessing expressive vocabulary and the other expressive grammar. While the P‐ITPA manual classifies children as impaired if their language abilities fall one SD or more below the mean, our study adopts a more conservative threshold. Based on the DELPHI study (Lüke et al. 2023), the consensus in German‐speaking countries defines DLD as performance 1.5 SD, or more below the mean on a standardized test. Therefore, in our study, children scoring at least 1.5 SD below the mean in either of the assessed language domains were classified as having language impairment consistent with risk for DLD.

### 2.3 Statistical Analyses

Correlations between the raw values were calculated to examine the relationships between the collected language data. Grammatical aspects from FRAKIS‐K were analysed separately from vocabulary scores since plural usage and word combination are separate criteria for being considered a late talker.

Next, a frequency analysis was conducted to determine how many children were identified with low expressive language at each measurement point, and the extent of overlap between these classifications. The primary criterion for language difficulties was language ability at age four. Children scoring at least 1.5 SD below the mean in at least one of the two P‐ITPA subtests were classified as having language impairment consistent with risk for DLD. Being a late talker in the SBE‐2‐KT and FRAKIS‐K assessments at age two served as predictors.

While the SBE‐2‐KT provides normative values for children being late talkers aged 23–24 months, children up to 25 months were included in the analysis to maximize the sample size. The FRAKIS‐K provides separate normative values for each month concerning the vocabulary, but suggests classification of two‐year‐olds as late talkers when meeting three different criteria (see above). Predictive validity of FRAKIS‐K was calculated separately for both, grammatical aspects included, as well as vocabulary alone.

The following metrics were calculated to assess the predictive validity: sensitivity, specificity, positive predictive value (PPV), negative predictive value (NPV), and relative improvement over chance (RIOC). The RIOC measures how much the hit rate improves beyond the chance level, making it useful for evaluating predictive validity (Marx and Lenhard 2010). RIOC values can be interpreted as following: below 33% as ‘unsatisfactory’, 33%–66% as ‘good’, and above 66% as ‘very good’ (Marx and Lenhard 2010).

Subsequently, receiver operating characteristic (ROC) analysis was performed to evaluate whether adjusting the cut‐off value could enhance predictive validity. The ROC curves visually represent the trade‐off between efficiency—highlighting the sensitivity and specificity values for different raw scores—and error rate across varying parameter values. A curve near the diagonal suggests random classification. The optimal cut‐off value was identified when both sensitivity and specificity reached their maximum values (Faller 2005). The aim was to achieve an RIOC of at least 66%, with special interest in increasing sensitivity.

For ROC analysis to determine the optimal cut‐off value, only the vocabulary score of FRAKIS‐K could be examined (without separate grammar criteria). While age‐specific normative data (cut‐off values) exist for each month, generating separate ROC curves for each age group would have resulted in small sample sizes, limiting statistical reliability. To ensure a sufficiently large sample, ideally with each vocabulary score represented by at least one child, the FRAKIS‐K vocabulary scores of children aged 23–25 months were analysed combined using normative values for 24‐month‐olds. Similarly, we had examined SBE‐2‐KT for children aged 23–25 months in one group, applying the normative values for 23–24‐month‐olds.

ROC curve analysis also yields the area under the curve (AUC), a measure used to compare diagnostic test performance (Faller 2005). The AUC represents the probability that a randomly selected unaffected child scores higher than a randomly selected affected child. As an indicator of classification accuracy, AUC values range from 0.5 to 1.0, with higher values reflecting better performance. Calculated using the trapezoidal method, AUC values are interpreted as follows (Greiner et al. 2000): below 0.5 = no predictive power; 0.5–0.7 = slight predictive ability; 0.7–0.9 = moderate predictive ability; 0.9–0.99 = high predictive ability; and 1.0 = perfect prediction. After adjusting the cut‐off values using ROC curves, predictive validity was recalculated and compared to evaluate improvements in predictive accuracy.

## 3 Results

### 3.1 Correlation Analysis

As shown in Table 1, the raw scores of the SBE‐2‐KT, FRAKIS‐K, and the two P‐ITPA subtests (vocabulary and grammar) exhibited highly significant positive correlations (*p* < 0.001). A strong correlation was found between the FRAKIS‐K vocabulary and SBE‐2‐KT. The correlation between P‐ITPA vocabulary and P‐ITPA grammar was moderately strong. The FRAKIS‐K vocabulary and SBE‐2‐KT at age two showed weak correlations with P‐ITPA vocabulary scores but moderately strong correlations with P‐ITPA grammar scores at age four.

**TABLE 1:** Pearson correlations of the raw values.

Variable	*n*	*M*	*SD*	1	2	3	4	5	6
**1**. SBE‐2‐KT (23‐25mths)	166	42.84	17.20	—					
**2**. FRAKIS‐K vocabulary	186	40.27	27.67	0.843^***^	—				
**3**. FRAKIS‐K usage of plural	183			0.533^***^	0.662^***^	—			
**4**. FRAKIS‐K usage of word combination	179			0.797^***^	0.628^***^	0.409^***^	—		
**5**. P‐ITPA vocabulary	145	24.48	9.59	0.284^***^	0.279^***^	0.282^***^	0.293^***^	—	
**6**. P‐ITPA grammar	145	21.84	9.27	0.438^***^	0.497^***^	0.467^***^	0.317^***^	0.608^***^	—

*** *p* < 0.001 (two‐sidedly).

### 3.2 Predictive Validity of FRAKIS‐K and SBE‐2‐KT

Table 2 provides an overview of the predictive validity values. Additionally, it presents the frequency distributions of children classified as having language impairment consistent with risk for DLD at age four or having a typical language (TL). Based on the reference test P‐ITPA, 8 out of 145 four‐year‐olds were identified with having risk for DLD, corresponding to a prevalence of 5.5%. In the SBE‐2‐KT group, 17 of 130 children were classified as late talkers. In the FRAKIS‐K (including grammar‐related criteria), 13 out of 143 children were classified as late talkers. In the FRAKIS‐K vocabulary, 17 of 145 children were identified as having language delay.

**TABLE 2:** Predictive validity values of SBE‐2‐KT, FRAKIS‐K (incl. grammar) and FRALKIS‐K vocabulary for having language impairment consistent with risk for DLD at age four (‐1.5 SD in P‐ITPA vocabulary and/or grammar).

		SBE‐2‐KT (23‐25 months)		FRAKIS‐K (incl. grammar)		FRAKIS‐K voc.	
		[95% Wilson CI]		[95% Wilson CI]		[95% Wilson CI]	
		TL	LT	TL	LT	TL	LT
P‐ITPA voc./gr.	TL	110	13	125	10	125	12
	risk for DLD	3	4	5	3	3	5
Sensitivity		0.57 [0.25 ‐ 0.84]		0.38 [0.14 ‐ 0.69]		0.63 [0.31 ‐ 0.86]	
Specificity		0.89 [0.83 ‐ 0.94]		0.93 [0.87 ‐ 0.96]		0.91 [0.85 ‐ 0.95]	
PPV		0.24		0.23		0.29	
NPV		0.97		0.96		0.98	
RIOC		0.51		0.31		0.58	
Prevalence at age 2;0 (predictor)		0.13		0.09		0.12	
Prevalence at age 4;0 (criterion)		0.05		0.06		0.06	
*n*		130		143		145	

Abbreviations: CI, confidence interval; LT, late talker; TL, typical language.

Predictive validity was based on language impairment consistent with risk for DLD assessed at the age of four. The SBE‐2‐KT demonstrated moderate sensitivity and high specificity. The PPV was low, while the NPV was high. The RIOC fell within the range that is considered good (33%–66%; Marx and Lenhard 2010).

For FRAKIS‐K (including grammar‐related criteria), the sensitivity was lower than that of SBE‐2‐KT. The specificity, PPV, and NPV were similar to those of SBE‐2‐KT. However, the RIOC was low, rendering it unsatisfactory.

For the FRAKIS‐K vocabulary, sensitivity, specificity, PPV, and NPV were slightly higher than those of SBE‐2‐KT. Again, the RIOC was moderate, which was considered good.

### 3.3 ROC Analysis and Adjusted Cut‐Off Values

**FIGURE 1:**
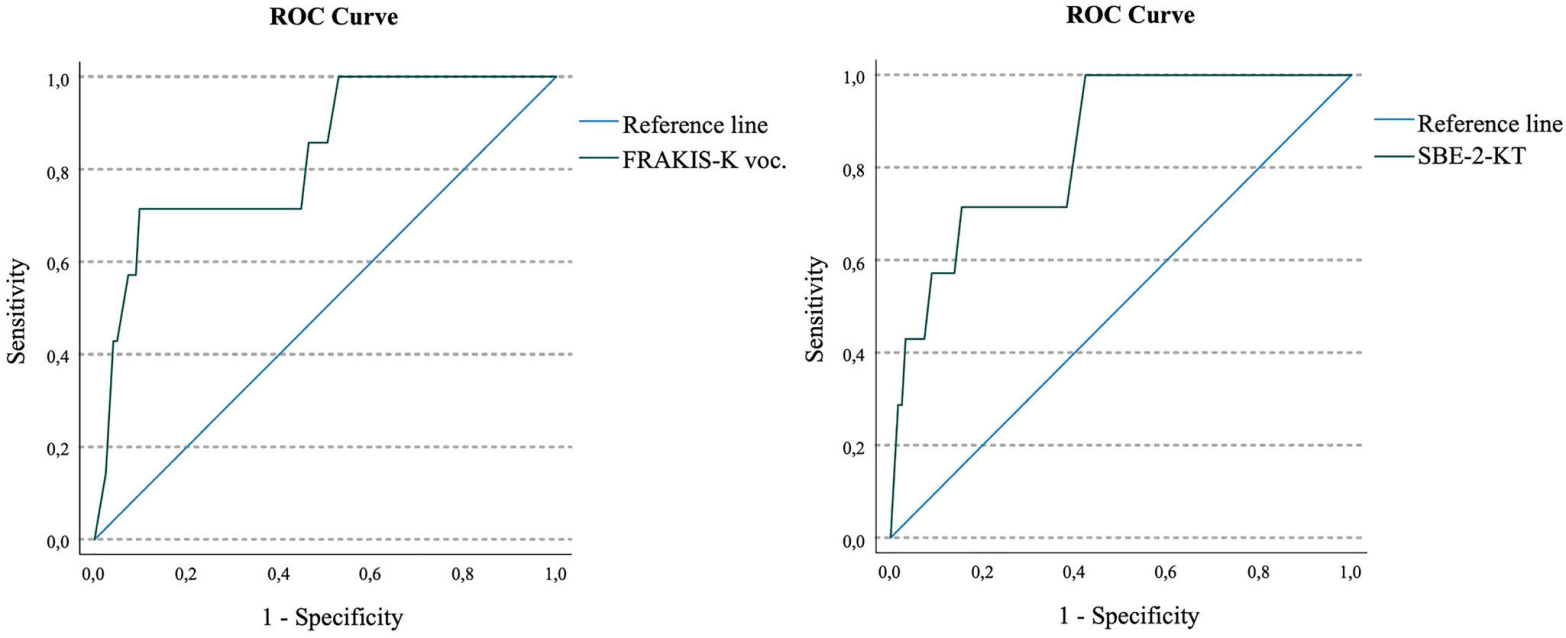
ROC analyses of FRAKIS‐K vocabulary and SBE‐2‐KT to identify risk for DLD at age four.

ROC analysis showed a moderate predictive power for both SBE‐2‐KT (*AUC* = 0.85; 95% CI [0.72, 0.97]) and FRAKIS‐K (*AUC* = 0.83; 95% CI [0.67, 0.98]). Figure 1 shows both ROC curves. The angle closest to 1 on the y‐axis and 0 on the x‐axis represents the best classifier (cut‐off).

For FRAKIS‐K, there was clearly one best option: the prevailing cut‐off (5 spoken words out of 102) showed the highest prediction (see Table 3). Note that this was the FRAKIS‐K norm for 24‐month‐olds, and the examination was for children 23–25 months old. The target of a RIOC > 66%, which represents very good predictive validity (Marx and Lenhard 2010), was achieved. Sensitivity, specificity, PPV, and NPV showed the highest values compared to all other calculations presented in this article.

**TABLE 3:** Predictive validity for adjusted cut‐off values of SBE‐2‐KT and FRAKIS‐K vocabulary (*n* = 130; prevalence of 0.54 for language impairment consistent with risk for DLD at age four).

		SBE‐2‐KT (23–25 months)								FRAKIS‐K voc. (23–25 months)	
Cut‐off values		18 (prevailing)		13		28		49		5 (prevailing)	
		TL	LT	TL	LT	TL	LT	TL	LT	TL	LT
P‐ITPA voc./gr.	TL	110	13	112	11	104	19	71	52	111	12
	risk for DLD	3	4	3	4	2	5	0	7	2	5
Sensitivity		0.57		0.57		0.71		1		0.71	
Specificity		0.89		0.91		0.85		0.58		0.90	
PPV		0.24		0.27		0.21		0.12		0.29	
NPV		0.97		0.97		0.98		1		0.98	
RIOC		0.51		0.52		0.65		1		0.67	
Prevalence at age 2;0 (predictor)		0.13		0.12		0.19		0.40		0.13	

Abbreviations: LT, late talker; TL, typical language.

Regarding SBE‐2‐KT, several cut‐off values could be discussed. A lower cut‐off (13 out of 58 spoken words) instead of the predominant cut‐off (18) would identify as many children who later developed language impairment (sensitivity) and showed similar but slightly better predictive values for specificity, PPV, and RIOC. However, a higher cut‐off (28) than the prevailing cut‐off would identify one more child who later developed language impairment, significantly improving sensitivity and RIOC but decreasing specificity and PPV. Table 3 also shows the possibility of identifying all seven cases of language impairment in the early screening with a cut‐off of 49 (out of 58) in SBE‐2‐KT. While sensitivity, NPV, and RIOC would be 100%, specificity and PPV would decrease immensely due to overidentification.

## 4 Discussion

Early identification of DLD within German‐speaking contexts remains a developing area with regard to validated predictive frameworks. This study aimed to contribute to that effort by evaluating the predictive validity of two standardized German parent‐report instruments, the FRAKIS‐K (Szagun et al. 2009, 2023) and the SBE‐2‐KT (Suchodoletz and Sachse 2009), when used at age two to identify language impairment consistent with risk for DLD diagnosed through standardized testing at age four. A secondary focus was placed on refining these tools by assessing the impact of different screening cut‐off values on predictive performance. The findings are intended to inform best practices for early identification and support the development of evidence‐based screening protocols within German paediatric care.

The results indicated that SBE‐2‐KT demonstrated good predictive power (*RIOC* = 0.51), with a sensitivity of 57% and a specificity of 89%. The ROC curve suggested moderate predictive power (*AUC* = 0.846). Adjusting the cut‐off value from 18 to 28 was associated with an increase in sensitivity to 71%, while specificity decreased slightly to 85%. However, given the very small number of children with DLD in the sample, these ROC‐based estimates should be interpreted with caution. The apparent improvement in predictive performance should be considered exploratory, as the identification of an optimal cut‐off value requires a substantially larger number of positive cases to yield stable estimates.

Regarding FRAKIS‐K, including the grammar‐related criteria, the results indicated unsatisfactory predictive power (*RIOC* = 0.31), with a sensitivity of 38% and a specificity of 93%. However, when considering only the vocabulary component of FRAKIS‐K, the predictive power improved significantly (*RIOC* = 0.58), with a sensitivity of 63% and a specificity of 91%. ROC analysis suggested a moderate predictive power (*AUC* = 0.83) for the FRAKIS‐K vocabulary of 23‐ to 25‐month‐old children assigned to the 24‐month‐old norm. The prevailing cut‐off value of 5 yielded a sensitivity of 71% and specificity of 90%, indicating very good predictive validity according to the RIOC. However, as with the SBE‐2‐KT, these estimates are based on a very small number of DLD cases and should therefore be interpreted as preliminary. Conclusions regarding the suitability of this or alternative cut‐off values require replication in larger samples.

Overall, comparison of the results to the predictive validity of parent reports on early language in other studies is limited because the methods, time between screening and testing, gold standards and samples used differ significantly. Compared to the study by Ullrich and Suchodoletz (2011) on the prognostic validity of the SBE‐2‐KT, it is noticeable that sensitivity, measured against the external criterion of a follow‐up screening one year later, is lower than in the standardized test conducted two years later (current study). Conversely, Ullrich and Suchodoletz (2011) reported significantly higher PPV. Since another parent questionnaire was used for follow‐up, method variance might be the reason for higher PPV.

Regarding the impact of DLD (Bishop et al. 2017), high sensitivity of an assessment tool is crucial. However, early identification of children developing DLD remains challenging because of the variability in early language acquisition (Pinstock and Schramm 2026) and the fact that some children with DLD are not late talkers (Snowling et al. 2016). Given these factors, a sensitivity of 71% appears acceptable. This threshold was met for both examined parent reports after adjusting the cut‐off values. Increasing sensitivity further would have resulted in a high rate of over‐identification, potentially causing unnecessary anxiety and misallocation of resources. Furthermore, a high NPV as well as low PPV was identified for both examined parent questionnaires regardless of the cut‐off values. The high NPV indicates that these tools are reliable for ruling out children not at risk, which supports their utility in large‐scale screening contexts. The PPV ranged from approximately 0.23 to 0.29, indicating that only about one quarter of children who screened positive met the study's criterion for language impairment consistent with risk for DLD at age four. This pattern is typical for screening tools applied to low‐prevalence conditions. However, it has important implications for interpretation. Specifically, the findings suggest that the primary strength of these instruments lies in their ability to rule out risk rather than to accurately identify affected children. These characteristics underscore the role of the parent reports as effective first‐tier screening tools that should be followed by more comprehensive assessments to confirm diagnoses and guide intervention planning rather than to provide definitive classification. This interpretation aligns with the two‐stage screening approach proposed by Holzinger et al. (2022), in which parent‐reported measures of expressive language, and parental concerns of two‐year‐old children are used as an initial step, followed by a targeted direct assessment of language comprehension for children who screen positive. This sequential procedure has been shown to achieve good diagnostic accuracy while improving efficiency by restricting more resource‐intensive assessments to at‐risk children, with direct assessment adding incremental predictive value beyond parent report alone (Holzinger et al. 2022). According to guidelines (AWMF 2022), this tiered approach is already reflected in current practice in Germany, where language screening during the U7 paediatric check‐up is intended to identify children in need of follow‐up rather than to provide a definitive diagnosis. This distinction between ruling out and confirming risk is central to the interpretation of screening outcomes. In the present study, the predictive profile of both instruments is characterized by high confidence in negative results but limited precision in positive classifications, which is consistent with their intended role in early detection frameworks.

While the prevalence of language impairment consistent with risk for DLD at age four was 0.54, 13% of 23‐ to 25‐month‐olds were classified as late talkers based on FRAKIS‐K vocabulary, and 19% based on SBE‐2‐KT. These findings align with those of Klee et al. (2000), who estimated a 5% prevalence of DLD and 15% screening positivity rate.

Regarding the predictive validity of the examined parent reports using the given thresholds, the FRAKIS‐K—including the grammar‐related criterion—demonstrated unsatisfactory predictive validity. Children were classified as late talkers at age two if their vocabulary fell within the lowest percentile, they did not combine words, and they did not use plural forms. When only considering the aspect of vocabulary in the FRAKIS‐K, predictive validity appeared to improve compared to the full set of criteria including grammatical indicators. However, this observation should be interpreted with caution. While it may suggest that vocabulary is a particularly informative early indicator, it does not necessarily imply that excluding grammatical criteria improves the instrument in a generalizable way. Many studies have identified early word production as one of the most reliable predictors of later language deficits (Sansavini et al. 2021). Therefore, not applying the additional criterion but vocabulary only when identifying late talkers using FRAKIS‐K might be beneficial. (Another option would be to account for the aspects of combining words and using plural forms in the same way as knowing another word in the checklist instead of defining separate criteria.) Due to the low incidence of language impairment in the current sample, the assumption requires further investigation with a larger dataset. In this context, the observed pattern in the present study may reflect the strong predictive role of early vocabulary; however, given the structural differences between instruments, this interpretation remains tentative.

FRAKIS‐K classifies children as having language delay when their vocabulary score falls within the lowest percentile of the normative sample (Szagun et al. 2023). In the current study, 12% of the sample was identified as having language delay. However, when combining the age groups of 23 to 25 months and applying the norm for 24‐month‐old children, the predictive validity improved significantly, with 13% of the sample classified as having language delay. This might be an indication that the threshold for identifying late talkers in the vocabulary of FRAKIS‐K could be set higher. To better identify children who may later develop DLD, the proportion of late talkers at an early age should exceed 10% (Klee et al. 2000). Given the gold standard used in this study, a recalibration of the FRAKIS‐K might be beneficial. However, the limited number of children with DLD in the sample substantially constrains the ability to draw firm conclusions about optimal cut‐off values. Although the findings suggest that a higher threshold may improve identification, these results should be considered exploratory. Robust evaluation of alternative cut‐offs requires replication in larger samples with a sufficient number of affected children.

In contrast, SBE‐2‐KT classifies children as late talkers if their vocabulary scores fall within the lowest 14%. In our sample, 13% of two‐year‐old children met this criterion. It is important to note that the examined sample included children aged from 23 to 25 months to increase the sample size, while normative data applies to children aged 23 to 24 months. (In our sample, 25 months referred to a maximum age of 25 months and 15 days.) Predictive validity improved when the cut‐off value was set higher, resulting in a new prevalence of 19% in late talkers, although this adjustment also increased the number of false positives. A closer look reveals that adjusting the cut‐off value ultimately results in only one additional child being correctly identified. This reflects the very small number of children with DLD in the sample, whereby changes in classification are driven by only a few individual cases, further underscoring the instability of sensitivity estimates. In this context, ROC analyses used to derive alternative cut‐off values are inherently unstable, as they rely on very few positive cases. As a result, the observed changes in sensitivity and specificity should not be interpreted as definitive evidence for improved thresholds but rather as exploratory indications. Any recommendations for adapting the SBE‐2‐KT cut‐off therefore require confirmation in larger and more representative samples. Therefore, recommendations for adapting the SBE‐2‐KT are limited. However, the difference in the word size of the suggested cut‐off values between SBE‐2‐KT and FRAKIS‐K is striking. The word lists of FRAKIS‐K and SBE‐2‐KT differed significantly, with only eight overlapping words. Notably, one child with risk for DLD at age four expressively used only five words from FRAKIS‐K at age two but 28 words from SBE‐2‐KT. If the words included in the FRAKIS‐K offer better differentiation than those in the SBE‐2‐KT checklist cannot be concluded by the results of this study. A larger sample with more cases of DLD is required for a more detailed analysis. More generally, comparisons between FRAKIS‐K and SBE‐2‐KT should be interpreted with caution, as the instruments differ in several fundamental aspects. These include the size and composition of their vocabulary checklists, the normative thresholds used to define late talking, and the criteria applied for classification (e.g., inclusion of grammatical markers in FRAKIS‐K). Without a systematic analysis that isolates these factors, it is difficult to determine whether observed differences in predictive performance reflect true differences in predictive validity or are driven by differences in instrument design. Accordingly, the present findings do not allow firm conclusions regarding the relative superiority of one instrument or component over another but rather highlight potentially relevant features that warrant further investigation.

### 4.1 Limitations

This study has several limitations that should be considered when interpreting the findings. First, although a community sample was used to reduce spectrum bias, the overall sample size was relatively small. This was primarily due to the resource‐intensive follow‐up assessments conducted at four years of age. A strength of the design is that all participants were assessed at follow‐up, thereby avoiding verification bias.

A key limitation concerns the representativeness of the sample. Participants were recruited from a community in Hanover, and participation was voluntary, resulting in a sample skewed toward families with higher educational backgrounds. Most children had at least one parent with a university degree, and there was additional attrition of families with lower educational levels over time. Given that socioeconomic status is a well‐established factor associated with early language development and risk for DLD (Snowling et al. 2016), this sampling bias likely contributed to the low prevalence of language impairment observed in the study. As a result, children at higher risk for DLD may be underrepresented.

This low base rate has important implications for the interpretation of predictive validity. Predictive indices such as PPV and NPV are directly influenced by prevalence. In the present sample, the low prevalence of DLD likely contributed to relatively low PPV, potentially underestimating the screening tools’ ability to correctly identify affected children.

Relatedly, the very small number of children classified as having DLD at follow‐up (8 out of 145) limits the reliability of several key estimates. Measures such as sensitivity and AUC depend on the number of true cases and become unstable when case numbers are low. In this study, each individual case represents a substantial proportion of the ‘risk for DLD’ group, meaning that the inclusion or exclusion of a single child can markedly influence sensitivity estimates and ROC curves. Consequently, these indices should be interpreted with caution and may not generalize to larger or more representative samples.

The small number of cases also constrains the interpretation of analyses aimed at identifying optimal cut‐off values. Although ROC analyses are appropriate for evaluating diagnostic accuracy, they require sufficient numbers of positive cases to produce stable estimates. In the present study, the suggested cut‐off adjustments for the FRAKIS‐K and SBE‐2‐KT should therefore be considered exploratory and require replication in larger and more diverse samples.

In addition, the comparison between FRAKIS‐K and SBE‐2‐KT is limited by structural differences between the instruments, including variations in vocabulary content, checklist length, scoring procedures, and classification criteria. These differences complicate direct comparisons, making it difficult to attribute observed differences in predictive performance solely to differences in underlying predictive validity.

Finally, the choice of outcome criterion represents a further limitation. The use of a threshold of −1.5 SD in at least one subtest as an indicator of risk for DLD is subject to ongoing debate (Lüke et al. 2023). At the same time, clinical diagnosis cannot be considered a definitive gold standard due to the lack of universally accepted diagnostic criteria. This highlights a broader challenge in DLD research and should be taken into account when interpreting the study's findings.

### 4.2 Conclusion

In conclusion, the study's longitudinal design, clinical relevance, and comprehensive evaluation of predictive validity provide a strong foundation for interpreting the findings. The findings suggest that both the SBE‐2‐KT and FRAKIS‐K vocabulary assessments are valuable tools for the early detection of language development issues during the U7 preventive medical check‐up for two‐year‐old children in Germany. Their use could facilitate more precise monitoring and support through parental guidance (Neumann et al. 2024) or interventions in day‐care settings. Their primary utility lies in reliably identifying children unlikely to develop DLD, whereas positive results should be interpreted as indicators for further assessment rather than as evidence of disorder. However, any diagnostic recommendation should be approached cautiously, emphasizing language promotion rather than definitive labelling. Importantly, screening outcomes may be better interpreted within a graded rather than dichotomous framework. Differentiating levels of risk can guide proportionate follow‐up, with moderate‐risk children primarily benefiting from family‐centred language promotion, and high‐risk children requiring further assessment and possible referral (Holzinger et al. 2022). Such an approach may help balance early support with the avoidance of over‐referral and unnecessary parental concern. Further research is necessary to refine these assessments, particularly in at‐risk populations, to determine more accurate cut‐off points. Larger samples with a substantially higher number of children with DLD are essential to obtain stable and generalizable estimates of predictive validity, particularly for sensitivity and ROC‐based analyses. Additional variables beyond questionnaires may play a crucial role in language development. Insights from studies, such as the Victoria study (Eadie et al. 2022) highlight the need for a broader research framework, including similar investigations within the German context.

## Ethical Approval

The study was approved by the University Research Ethics Committee of the Leibnitz University of Hanover, Germany, on July 8th 2015 (project number: EV LUH 03/2015), and all parents provided written informed consent.

## Conflicts of Interest

Satyam Antonio Schramm co‐developed the FRAKIS‐K and received an author's fee from Pearson in the past. Eveline Pinstock is Satyam Antonio Schramm's research assistant.

## Data Availability

The datasets generated and analysed during the current study are available from the corresponding authors upon reasonable request.
